# Direct Transition from Triplet Excitons to Hybrid Light–Matter States via Triplet–Triplet Annihilation

**DOI:** 10.1021/jacs.1c02306

**Published:** 2021-05-11

**Authors:** Chen Ye, Suman Mallick, Manuel Hertzog, Markus Kowalewski, Karl Börjesson

**Affiliations:** † Department of Chemistry and Molecular Biology, University of Gothenburg, Kemigården 4, 412 96 Gothenburg, Sweden; ‡ Department of Physics, Stockholm University, Albanova University Centre, 106 91 Stockholm, Sweden

## Abstract

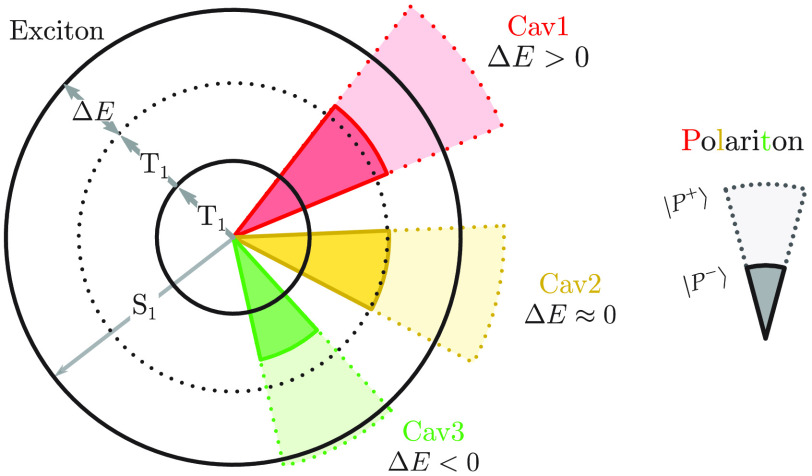

Strong light–matter coupling generates hybrid states that inherit properties of both light and matter, effectively allowing the modification of the molecular potential energy landscape. This phenomenon opens up a plethora of options for manipulating the properties of molecules, with a broad range of applications in photochemistry and photophysics. In this article, we use strong light–matter coupling to transform an endothermic triplet–triplet annihilation process into an exothermic one. The resulting gradual on–off photon upconversion experiment demonstrates a direct conversion between molecular states and hybrid light–matter states. Our study provides a direct evidence that energy can relax from nonresonant low energy molecular states directly into hybrid light–matter states and lays the groundwork for tunable photon upconversion systems that modify molecular properties in situ by optical cavities rather than with chemical modifications.

## Introduction

**Scheme 1:**
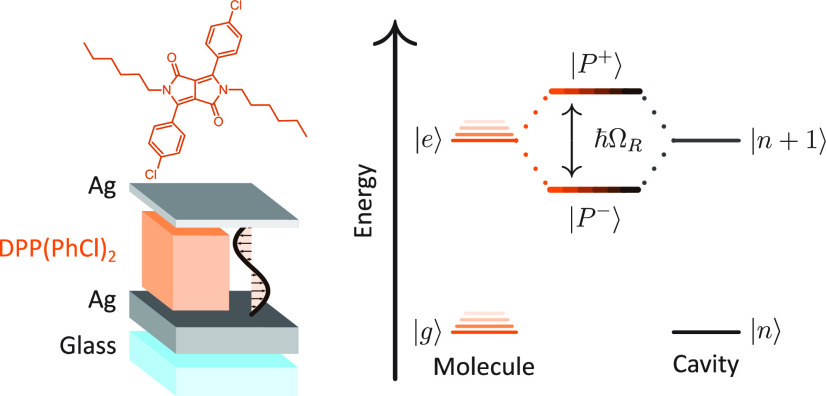
Molecular Structure of DPP(PhCl)_2_, Cavity Configuration, and Energetic Landscape of Molecular States under Strong Light–Matter Coupling

The interaction between light and matter has been a source of fascination since ancient times.^1^ Photons interact with the induced dipole of molecular transitions. When the strength of this interaction is larger than energy dissipation from the system, the strong coupling regime is reached. This phenomenon leads to the generation of light–matter hybrid states, called polaritons (or dressed states; Scheme 1). Polaritons are quasi-particles, with both photonic and excitonic contributions, and therefore inherit properties from both.^2,3^ The photonic characteristics imbue a delocalized character, while the excitonic characteristics enable interaction with optically inactive material states.^4,5^ Strong light–matter coupling and its associated unique properties can be accessed with passive optical cavity devices and have been recently used to achieve large advancements in optics,^6^ electronics,^7^ catalysis,^8^ and quantum devices.^9^

In the condensed phase, strong light–matter coupling was first achieved with inorganic semiconductors, which normally form loosely bound Wannier–Mott excitons.^10^ These have small exciton binding energies and a large wave function overlap, and their strong light–matter coupling properties have been intensively studied.^11^ In contrast, organic molecules normally form Frenkel excitons with large transition dipole moments and consequently an increased light–matter coupling. They also provide access to a richer variety of photochemical and photophysical processes and therefore offer a host of new research opportunities.^12,13^ Recently, the transition between polaritonic and molecular states has been studied by observing differences in rates,^14^ including energy transfer,^15,16^ reverse intersystem crossing,^17,18^ and triplet fusion/singlet fission.^19,20^

Among these excited-state transitions is triplet–triplet annihilation (TTA), which involves the upconversion of low energy photons into high energy ones.^21^ TTA is attractive, as it provides a generic method of boosting the maximal efficiency of a single junction photovoltaic device from 32 to 51%.^22,23^ TTA occurs when two excited triplet states (often called a triplet pair) interact when their energy level configuration meets a certain condition (*E*_S1_ – 2*E*_T1_ < 0).^24,25^ Molecular energy levels can be tuned to meet the correct conditions for TTA through molecular design and synthesis.^26^ However, this strategy is time-consuming and is limited by the availability of synthetic pathways and the laws of molecular photophysics. Intriguingly, Polak et al. recently found a connection between polaritonic and a quintet triplet pair (^5^(TT)) in geminate TTA. Such a connection between light–matter and molecular centered states opens the possibility to use strong light–matter interactions to tune the energetics within photon upconversion.^20^

In this article, we demonstrate the direct conversion of the excitonic triplet pair to hybrid light–matter states by turning an endothermic TTA process into an exothermic one. Initially, a TTA-unfavored molecule is strongly coupled to the vacuum field, where cavity polaritons with the correct energetics for exothermic TTA are created. Using temperature-resolved photoluminescence spectroscopy, we show that TTA is only observed at low temperatures when energetically accessible cavity polaritons are present. The TTA process was then modeled using time-resolved spectroscopy data to describe the new TTA pathway, which was opened in the strong coupling regime.

## Results

Aromatic molecules such as anthracene, pyrene, and perylene are typical TTA annihilators with long-lived triplet states and appropriate singlet–triplet energy alignments. The S_1_ state is generally less distorted than the T_1_ state compared to the ground state, and it is therefore more sensitive to substitution.^27^ Synthetic chemists have exploited this to develop a library of diketopyrrolopyrrole (DPP) annihilators with different S_1_ energies without perturbing the T_1_ energy.^26^ Here the same effect was achieved with strong coupling, where 3,6-bis(4-chlorobenzene)diketopyrrolopyrrole (DPP(PhCl)_2_, SI 1.1 and Figure S1) was coupled to the electromagnetic field of an optical cavity. Twice the T_1_ energy of DPP(PhCl)_2_ (1.15 eV) is slightly lower than the S_1_ energy (2.36 eV). This energy level alignment therefore makes TTA an endothermic process.^26,28,29^ After excitation to the Franck–Condon state, DPP(PhCl)_2_ molecules in solution quickly relax to the lowest vibrational level of the singlet excited state. In the pristine solid state, the absorption envelope is conserved although slightly red-shifted, which indicates a retained energy configuration (Figure S2). The energy of the Franck–Condon state, as well as the relaxed excited state, was determined in both solution and neat films for further discussion below.

**Figure 1:**
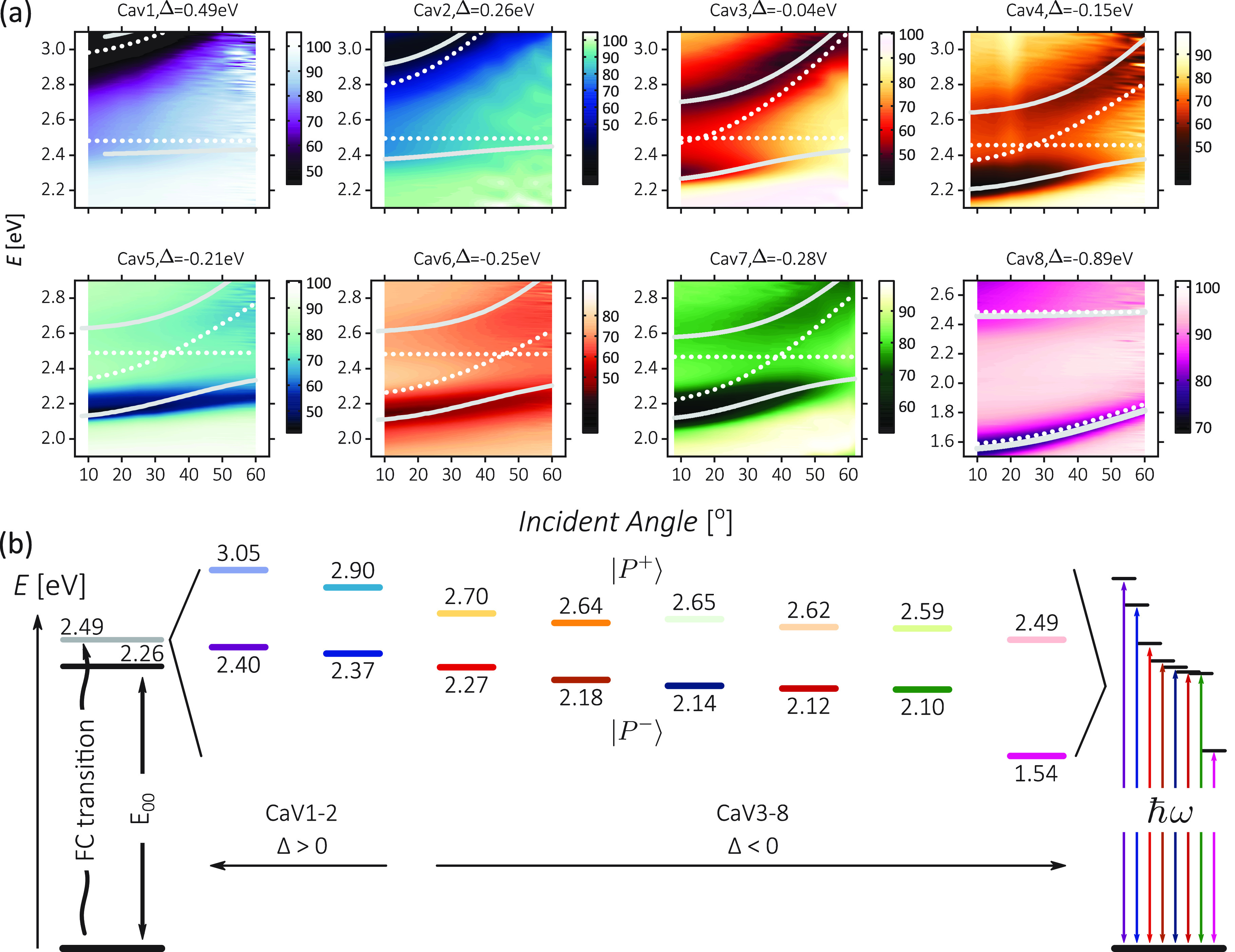
Characterizing the strong light–matter interaction. (a) Angle-resolved reflectivity of sensitizer/DPP(PhCl)_2_ cavities at different tunings together with a fit to a coupled oscillator model. (b) Energy configurations of DPP(PhCl)_2_ as a bare film and inside cavities at different detunings. The cavities with an energetic driving force for triplet–triplet annihilation (*E*_|P_^–^_⟩_ – 2*E*_T1_) are also indicated.

The large transition dipole moment of DPP(PhCl)_2_ enabled the collective strong light–matter coupling regime to be reached. Strong coupling was achieved by placing triplet sensitizer-doped DPP(PhCl)_2_ thin films within Fabry–Pérot cavities (Scheme 1, SI 1.2 and 1.3). The cavities consisted of a molecular film sandwiched between a 30 nm Ag top mirror and a 150 nm Ag bottom mirror. The optical cavity confined the electromagnetic field at the optical resonance, as dictated by the cavity thickness. When the optical resonance matched the molecular transition, the cavity and molecule became coupled (Scheme 1). The strong light–matter coupling was characterized as a function of cavity resonance tuning. Figure 1a shows the angle-resolved reflectivity of eight cavities (Cav1–8), which were tuned at different energies around the energy of the molecular exciton. In these cavities, the molecular absorption split into two polaritonic branches, |P^–^⟩ and |P^+^⟩. Both branches shifted to higher energies as the incident angle increased, but |P^–^⟩ never crossed the energy of the molecular exciton. The observed anticrossing is a typical feature of strong light–matter coupling. To extract the coupling strength, polariton energies, detuning, and composition, the dispersion of polariton energies was fitted to a Jaynes–Cummings type model (eq 1):
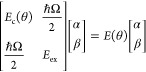
1where *E*_c_ is the cavity photon energy, which is related to the incident angle θ, *E*_ex_ is the exciton energy (the Franck–Condon state), and *ℏΩ* is the Rabi splitting. The in-plane distribution of polariton energies was obtained from the eigenvalues of the Hamiltonian. The Hopfield coefficients, |α|^2^ and |β|^2^, represent the fractional excitonic and photonic contributions to the corresponding polaritons; the cavity detuning is defined as the difference between the cavity energy and the molecular energy (Δ = *E*_c_ – *E*_ex_).^30^ The collective Rabi splitting (0.42–0.44 eV for all) was equal or larger than the full width at half-maximum (fwhm) of the molecular transition (0.42 eV), which indicates that the coupling strength was larger than the dissipated energy, and the strong coupling regime was reached. Furthermore, the relative coupling strength is 0.18, resulting in the system being on the border of the ultrastrong coupling regime (defined as a relative coupling strength larger than 0.2).

Strong light–matter coupling provides a possibility to tune the energy level alignment by splitting the Franck–Condon state into two polariton branches (Figure 1b). As the cavity resonance energy is decreasing, the |P^–^⟩ energy decreases below the energy of the relaxed exciton (*E*_00_) and also below two times the triplet energy. Furthermore, when the cavity resonance energy varies, the excitonic and photonic contribution to |P^–^⟩ also changes (Figure S3). By tuning the cavity resonance frequency to lower energies, the energy of |P^–^⟩ decreases, but the exciton fractional contribution to |P^–^⟩ also decreases. When the cavity energy is too low (Cav8), the connection between bare molecular states and polaritonic states diminish, limiting the practical tuning range.

We have so far demonstrated that the singlet excited state of DPP(PhCl)_2_ can be strongly coupled to the vacuum electromagnetic field. We will here show that the change in energy level alignment by the formation of polaritonic states has a profound effect on the system’s ability to perform TTA, giving large evidence for a direct pathway from the excitonic triplet pair to |P^–^⟩. As a triplet sensitizer, we chose platinum tetrabenzotetraphenylporphyrin (PtTBTP) at a doping concentration of 1% in otherwise pristine DPP(PhCl)_2_ films (SI 2.2, Figure S4). The high intersystem crossing efficiency and appropriate triplet energy (1.61 eV) makes PtTBTP a suitable triplet sensitizer for DPP(PhCl)_2_. The small amount of doped sensitizer is not coupled to the cavity due to the mismatch in resonance frequency. Pun et al. found that triplet DPP(PhCl)_2_ in solution is unable to undergo efficient TTA due to the endothermic nature of the process (Δ*E* = *E*_S1_ – 2*E*_T1_ = 60 meV).^28^ Thus, the DPP(PhCl)_2_ triplet relaxes through monomolecular intrinsic decay in solution. We examined the TTA behavior of PtTBTP/DPP(PhCl)_2_, PtTBTP, and DPP(PhCl)_2_ films under N_2_ atmosphere using a pulsed excitation source (λ_ex_ = 613 nm, Nd:YAG source). The phosphorescence lifetime of PtTBTP decreased from 17.6 μs in PtTBTP pristine films to 3.1 μs in PtTBTP/DPP(PhCl)_2_ films (Figure S5, see SI 2.3). That the phosphorescence lifetime of PtTBTP decreased by 1 order of magnitude in the presence of DPP(PhCl)_2_ suggests that efficient triplet–triplet energy transfer occurred from PtTBTP to DPP(PhCl)_2_. The reduced sensitizer emission in PtTBTP/DPP(PhCl)_2_ films was followed by weak molecular emission from DPP(PhCl)_2_, which exhibited the same spectral envelope as the directly excited pristine DPP(PhCl)_2_ film. The time-resolved emission showed that the emission lasts for a few hundred microseconds, which is approximately 5 orders of magnitude longer than the fluorescence lifetime (Figure S6). Furthermore, no emission from a pristine DPP(PhCl)_2_ film was observed when exciting at the absorption maximum of PtTBTP (λ_ex_ = 613 nm; Figure S7). We then inferred that DPP(PhCl)_2_ can perform TTA in the solid state under pulsed excitation conditions (10 Hz, Nd:YAG source).

The emission from PtTBTP/DPP(PhCl)_2_ films was examined in the strong coupling regime using the same setup. The cavities have different thicknesses, and therefore having different energy level alignment and material contribution to |P^–^⟩. Cav3–7 showed polariton emission when DPP(PhCl)_2_ was excited directly, where the dispersive emission envelope resembled the |P^–^⟩ absorption rather than the nondispersive bare molecular emission (Figure S9, SI 2.5). The polariton emission has a narrow line shape. This confined energy distribution is beneficial when used to excite a single bandgap device, which is a general aim within the field of TTA-UC.^31−33^ The lack of polariton emission from Cav1–2 was attributed to the higher energy of |P^–^⟩ as compared to the vibrationally relaxed excited singlet state (*E*_00_) of DPP(PhCl)_2_. Similarly, the large detuning observed in Cav8 disturbed the light–matter coupling.

**Figure 2:**
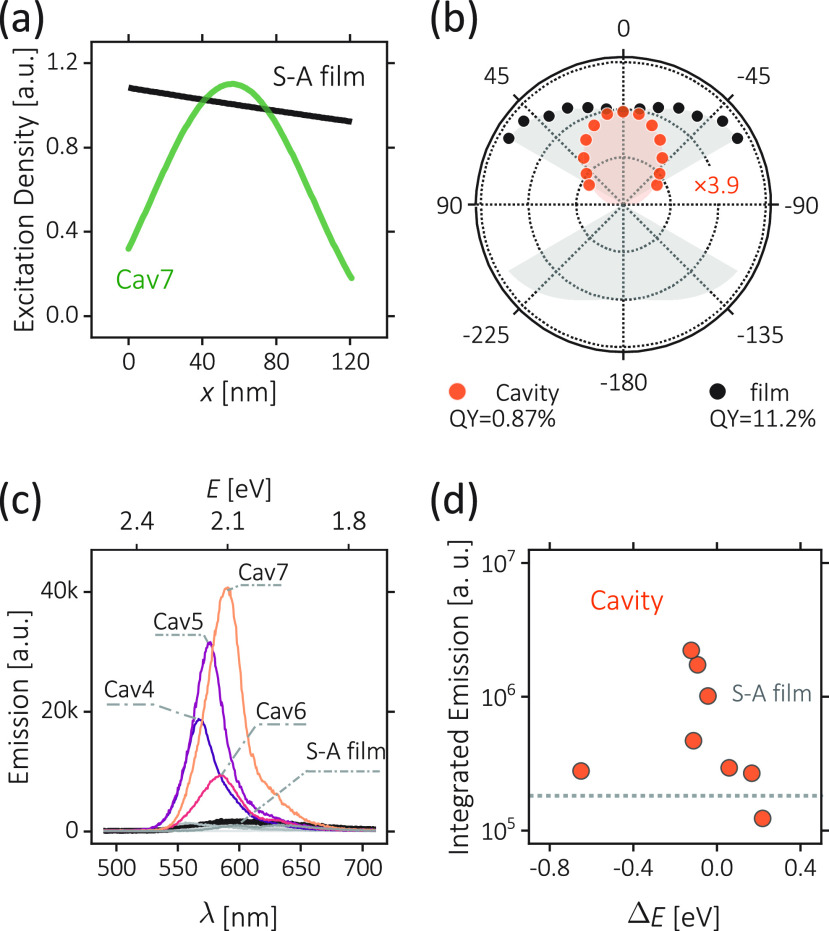
Room temperature TTA-UC emission. (a) Excitation density at 613 nm inside a sensitizer-doped film and a representative cavity (Cav7) as calculated by a transfer matrix approach using the same excitation power. (b) Angle-dependent emission intensity of DPP(PhCl)_2_ in film and cavity (same thickness as Cav7). The shadow areas represents the quantum yield. (c) Room temperature TTA-UC emission spectra of sensitizer-doped film and sensitizer-doped film in cavities, detected at normal configuration. (d) Correlations between integrated TTA-UC emission and energetic driving force for TTA (*E*_|P_^–^_⟩_ – 2*E*_T1_). The dotted line marks the TTA-UC emission from the sensitizer-doped film.

To correlate the strength of TTA-UC between films and cavities, the sensitizer absorption and polariton emission quantum yields were first examined. Nonresonant excitation in a Fabry–Pérot cavity generally lowers the amount of photons absorbed as compared to a bare film.^34,35^ The excitation profile inside the cavity and in a bare film was simulated using a transfer matrix approach (Figure 2a, Figure S10).^36−38^ This showed a similar maximal excitation density inside the cavity, with some distribution effects, under the same excitation conditions. The emission quantum yield normalized angle resolved polariton emission is shown in Figure 2b. The polariton emission is highly angle dependent and much weaker compared to the strong emission from a bare film. In the coming sections, all measurements are performed in a normal configuration. Thus, for the same concentration of excited states, we would expect the bare film to emit about 3.9 times more intense compared to the cavity. We also excluded the Purcell effect as a reason for emission enhancement (SI 2.6).^39^

The same emission profiles for all cavities were displayed when the sensitizer was excited as compared to direct excitation. Furthermore, the oxygen sensitivity indicated that the photoluminescence originated from TTA when exciting the sensitizer (SI 2.7, Figure S12). Figure 2c shows the integrated TTA-UC emission from all cavities and a sensitizer-doped film (S-A film). The emission from Cav1 is lower as compared to the bare film. With more negative detuning, the energetic requirements for exothermic TTA is fulfilled. Now the intensity of emission is increased dramatically, up to 9 times higher compared to the sensitizer-doped film, this despite the similar density of excited sensitizers and the lower emission quantum yield of the annihilator. Thus, when changing the cavity detuning, which increases the energetic driving force for TTA, we observed a gradual “turn on” of TTA. However, at a very large detuning (Cav8), the emission intensity is again similar to that of a neat film, indicating that it is not only an energetic driving force for TTA that is of importance, but also that the detuning must not be too large. We propose that the TTA efficiencies will be affected by multiple cavity parameters, including the excitonic/photonic fraction, and the TTA energy gap. The photonic fraction will affect the emission efficiency and the excitonic contribution could affect the transition efficiency. The energy gap showed the strongest correlation with TTA efficiency in the DPP(PhCl)_2_ cavities (Figure 2d).

**Figure 3:**
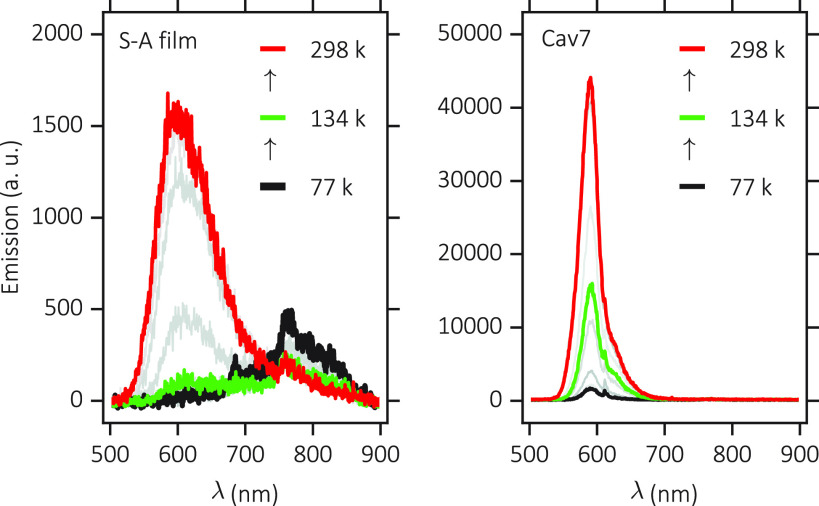
Temperature dependence of TTA-UC emission. Sensitized TTA emission at different temperatures for the sensitizer-doped film (left) and Cav7 (right) after 1 μs delay (*λ*_ex_ = 613 nm, *I*_ex_ = 0.19–0.23 mJ/pulse).

Next, the different TTA properties observed for the sensitizer-doped film and Cav7 at different temperatures were investigated to confirm that triplet excitons were converted by TTA directly to light–matter states. For the doped film, the emission is weak and highly temperature dependent, such that it is undetectable below ca. 130 K (using an ICCD detector; Figure 3). At room temperature, the upconverted emission from Cav4–7 was 1 order of magnitude stronger than that from the the doped film and other cavities. Among the strongly emissive cavities, Cav7 has the lowest |P^–^⟩ energy and therefore the largest potential for exothermic upconversion (Figure 2d). The sensitized TTA |P^–^⟩ emission from Cav7 showed more intense emission at all temperatures than the bare film. TTA was detectable in Cav7, even at temperatures from 77 to 130 K, while the doped bare film exhibited no apparent emission at these temperatures. The large emission at low temperatures from Cav7 indicates a direct upconversion pathway from the triplet pair to the polaritonic state in cavities having an exothermic TTA energy alignment, like Cav7.

**Figure 4:**
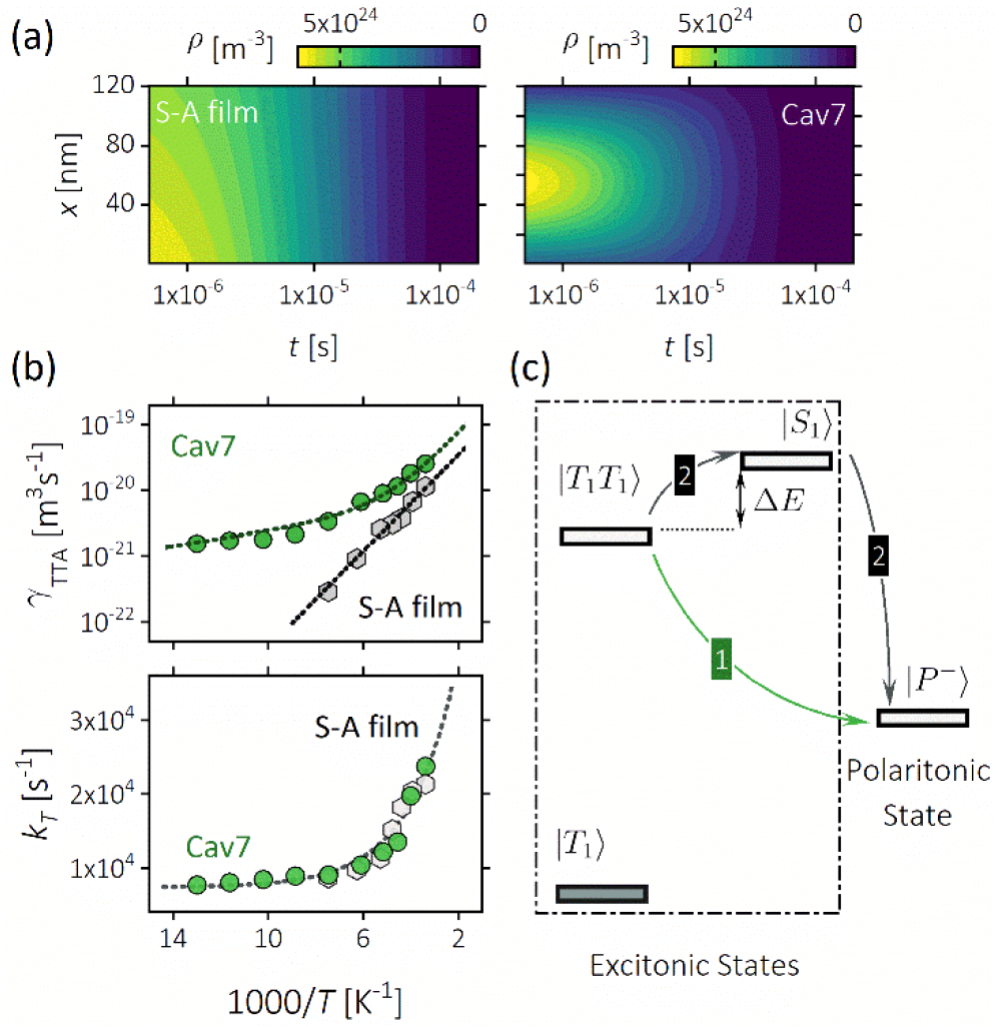
Dynamics of TTA-UC emission. (a) Simulated density distribution of triplet DPP(PhCl)_2_ over time and position at room temperature. The set time scale is relevant for fitted decay kinetics. (b) Calculated TTA kinetic parameters of DPP(PhCl)_2_ inside (Cav7) and outside (sensitizer-doped film) a cavity. (c) Exciton to polariton energy pathway.

To further investigate the mechanism of TTA starting from sensitizer excitation, time-resolved photoluminescence analysis was performed, and a corresponding kinetic model was developed. TTA in the solid state requires a close interaction between two triplet excitons (a triplet pair) and thus involves exciton diffusion through the solid matrix.^40^ Recently the intermediate triplet pair states have become the focus of several studies, which greatly promotes the kinetic analysis of geminate pair interaction in singlet fission and triplet fusion.^41−43^ Here we chose the classic model, which treats the triplet pair species as a transition state.^44,45^ The TTA kinetics can therefore be described by the following equation (eq 2):^46^
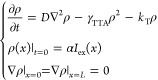
2where ρ is the density of triplet excitons, *D* is the diffusion coefficient of triplet excitons, *γ*_TTA_ is the annihilation rate constant in solid state, and *k*_T_ is the intrinsic decay constant of triplet excitons. The initial density of triplet excitons after excitation is proportional to the excitation intensity *I*_ex_ at position *x*, and *L* is the film thickness. The initial contributions along the cavity depth was calculated by a transfer-matrix approach (see SI 1.6 for details). The kinetic equation (eq 2) can be numerically solved (Figure 4a), with the overall delayed fluorescence as an indicator for the process (eq 3):
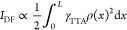
3where *I*_DF_ is the intensity of delayed fluorescence.^47,48^ Transient absorption of the doped bare film was recorded to calculate the initial density of triplet DPP(PhCl)_2_ after laser excitation (SI 2.8, Figures S13–S15). The time evolution of TTA emission of doped films inside (Cav7) and outside a cavity was then monitored at different temperatures and excitation intensities (SI 2.9, Figures S16 and S17). The fitted data of rate constants indicated that triplet excitons have the same intrinsic decay lifetime inside and outside the cavity (Figure 4b), and therefore that the triplet exciton itself was not affected by the vacuum field inside the cavity. This was expected, as triplet excitons have a very small transition dipole moment and the cavity was not tuned to the energy of the triplet state. To fit the temperature dependence of the TTA rate accurately, two different TTA channels were needed, which covers the temperature dependence of both exciton diffusion and endothermic TTA (Figure 4c):
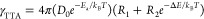
4where *D*_0_ is the intrinsic diffusion coefficient of triplet DPP(PhCl)_2_, which is dependent on the diffusion activation energy *E*_a_ and temperature, *k*_B_ is the Boltzmann constant, *T* is the temperature, and *R*_1_ and *R*_2_ are the TTA interaction radius of exciton-to-polariton and exciton-to-exciton TTA, respectively. The TTA interaction radius reflects the annihilation reaction intensity of the triplet pair, which is explained in Smoluchowski’s theory.^49−51^ We assumed that the triplet exciton diffusion is the same inside and outside cavities. This is a valid assumption because of the low transition dipole moment of the ground state to triplet transition and because of the triplet energy being much lower as compared to the cavity resonance. The diffusion activation energy was fitted to 12 meV, which is of comparable magnitude to reported values of triplet exciton diffusion in the solid state.^40,52−54^

The exciton-to-exciton TTA rate constant of DPP(PhCl)_2_ followed an Arrhenius-type behavior with temperature (Figure 4b). From this, we confirmed that TTA in doped films is an endothermic process with a calculated energy gap (Δ*E = E*_S1_ – 2*E*_T1_) of 67 meV. This value is close to that previously observed in solution (60 meV).^26^ The room temperature TTA rate constant of DPP(PhCl)_2_ is at a magnitude of 10^–20^ m^3^ s^–1^, which is much smaller than that of widely used TTA annihilators such as perylene and anthracene.^46,55,56^ The TTA rate constant drops dramatically with decreasing temperature, making the TTA-UC emission undetectable at low temperatures. In summary, the rate of exciton-to-exciton TTA of DPP(PhCl)_2_ in doped films is limited due to its endothermic nature.

By coupling the exciton to the electromagnetic field, hybrid light–matter states with the ability for exothermic TTA are created (Figure 1b). The TTA rate constant in Cav7 (2.6 × 10^–20^ m^3^ s^–1^) was larger than that of the sensitizer-doped film, because it is barrier free. However, the value is still smaller than that of the current solid state TTA-UC devices (10^–19^ to 10^–18^ m^3^ s^–1^).^57,58^ This can be explained by a reduced wave function overlap. The polaritonic states are delocalized, while the excitonic triplet states are localized in space.^59,60^ The difference in TTA rate constant between sensitizer-doped film and the cavity becomes more significant at low temperatures (Figure 4b). At low temperatures, upconversion only occurred directly to the low energy polaritonic state, whereas at higher energies, exciton-to-exciton upconversion followed by energy relaxation to |P^–^⟩ starts to contribute (Figure S18).

We have shown that triplet annihilation can occur directly to polaritonic states. We now turn our attention to the spin of the polaritonic state, as to assess if the increased rate of TTA is due to spin statistics. Triplet states can have three different values of their spin component, resulting in nine possible combinations for the triplet pair (SI 2.10, Scheme S3). Of these combinations are one triplet pair being an overall singlet encounter complex ^1^(TT), three triplet pairs being an overall triplet encounter complex ^3^(TT), and five triplet pairs being an overall quintet encounter complex ^5^(TT).^61,62^ It is assumed that the sample is free of an external magnetic field, the observed product states (S_0_ + S_1_) stem from the singlet state, and that spin–orbit coupling in the product is slow compared to fluorescence and polariton decay. Under these assumptions, the total angular momentum ***J*** must be conserved, and only the triplet pair with an overall singlet character will produce an excited singlet state by TTA.
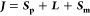
5where ***S*_p_** is the spin (or helicity) of the photons in the cavity, ***L*** is the molecular orbital angular momentum of the product state, and ***S*_m_** is the spin angular momentum of the molecule. In the strong-coupling regime, the cavity couples to the S_0_ to S_1_ transition of DPP(PhCl)_2_, which corresponds to an exchange of a single cavity photon with the molecular ensemble. The polariton states can be written in terms of Fock states and molecular electronic states:
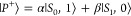
6
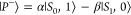
7where |0⟩ and |1⟩ are the zero- and one-photon Fock states of the cavity, respectively. Here we can assume that the vacuum state |0⟩ of the cavity contributes no spin (|***S*_p_**|=0), and the one photon state |1⟩ contributes one spin quantum (|***S*_p_**|=1). A closed shell molecular ground state has ***L*** = 0 and a dipole allowed, bright S_1_ state has |***L***| = 1 (π–π* transition for DPP(PhCl)_2_). Therefore, both polariton states must have |***J***| = 1 to conserve the total angular momentum. In conclusion, the TTA process is spin allowed from the triplet pair of singlet character to both polariton states. The spin statistics of TTA is thus not influenced by the formation of polaritonic states. It should be noted that there is significant experimental evidence of a loosening of the rule of spin conversation in the strong coupling regime, allowing for a slow but detectable direct route from a triplet pair of an overall quintet spin to polaritons. However, the slow rates of such a spin-unfavored process will have a negligible contribution to the total emission in sensitized TTA systems, such as the one studied here. This is because they will be kinetically outcompeted by faster ones in a system with rapid dynamics between free triplets and triplet pairs of various overall spins. Furthermore, dark states with different spin angular momentums can also contribute to the formation of the final polaritonic state.^20^ However, the rates of these spin-unfavored processes will be restricted and will have less contribution in a sensitized TTA system.^63^

We note here that recent theoretical predictions have shown that the ultrafast decay of the intracavity photon may play a significant role in facilitating cavity-mediated photochemical processes.^64−67^ Even though the overlap of a single triplet pair wave function with the collective lower polariton state may be small, the population in |P^–^⟩ gets removed on a sub 100 fs time scale by means of photon decay. It can thus be assumed that the back-reaction becomes sufficiently suppressed, counteracting the low wave function overlap.^59^ We therefore speculate that a large part of the emission enhancement seen in this study is due to the rapid decay of the |P^–^⟩ state, which shifts the equilibrium of the ^1^(T_1_T_1_) state toward the free S_0_ states.

## Discussion

We have demonstrated that hybrid light matter states can be populated directly from triplet states via triplet–triplet annihilation. By doing so, TTA can be activated from an otherwise TTA-inactive molecule. Our observations show unambiguously that triplet excitons can be converted directly into polaritons without any intermediate steps. The polariton states have an energy that is less than two times the triplet exciton energy, thus changing the TTA mechanism from an endothermic to an exothermic one. The temperature-dependent TTA kinetics data revealed that the triplet exciton itself is not coupled to the optical cavity, but the triplet pair from two triplet excitons could directly convert into exciton-polaritons by TTA. At low temperature, exciton-to-polariton TTA dominates, but with increasing temperature, exciton-to-exciton TTA starts to contribute. The easy tunability of optical nanocavities makes strong coupling especially versatile for modifying fundamental properties of molecular systems. We anticipate that the principles presented in this article will be widely applied in modern molecular science as a tool for building easily tunable systems and used to improve organic solar cells and other photoelectric devices.

## Supporting Information Available

Experimental section, supplementary figures, supplementary tables, and supplementary references (PDF)
