# Supplementary material for: Direct Transition from Triplet Excitons to Hybrid Light–Matter States via Triplet–Triplet Annihilation
Source: J Am Chem Soc. 2021 May 11;143(19):7501–8. doi: 10.1021/jacs.1c02306 (PMC8154526; doi:10.1021/jacs.1c02306)
Supplement: Supplementary file 1 — ja1c02306_si_001.pdf [file ja1c02306_si_001.pdf]

Supporting Information

Direct Transition from Triplet Excitons to Hybrid Light-Matter States via Triplet-Triplet Annihilation

Chen Ye[a], Suman Mallick[a], Manuel Hertzog[a], Markus Kowalewski[b] and Karl Börjesson[a]*

[a] Dr. C. Ye, Dr. S. Mallick, Dr. M. Hertzog, Dr. K. Börjesson
Department of Chemistry and Molecular Biology, University of Gothenburg, Kemigården 4, 412 96 Gothenburg (Sweden)
[b] Dr. M. Kowalewski
Department of Physics, Stockholm University, Albanova University Centre, 106 91 Stockholm (Sweden)
E-mail: karl.borjesson@gu.se

Table of Contents

1 Experimental Section 4

1.1 Synthesis of the annihilator 4

Scheme S1. Synthetic procedure of $DPP(PhCl)_2$. 4

Figure S1. 1H NMR spectrum of $DPP(PhCl)_2$. 5

Figure S2. Molecular photophysics from dilute solution to film. Normalized absorption and emission of 10^{-5} M $DPP(PhCl)_2$ in toluene solution, and in a pristine $DPP(PhCl)_2$ film. 6

1.2 Film preparation 6

1.3 Cavity preparation 7

Table S1. Parameters for the cavities in this work. 7

1.4 Modelling the angle-resolved reflectivity. 7

1.5 Steady state absorption, reflection and emission spectroscopy. 8

1.6 Transfer matrix method. 8

1.7 TTA photon upconversion emission. 10

1.8 Time resolved photoluminescence spectroscopy. 11

1.9 Transient absorption spectroscopy. 11

2 Supporting Experimental Results 12

2.1 Properties of cavities 12

Figure S3. Hopfield coefficient of $|P^-\rangle$ of the cavities. 12

2.2 Information of the sensitizer 12

Figure S4. Molecular structure and absorption and emission spectrum of PtTBTP in toluene. ... 12

2.3 Triplet-triplet energy transfer 13

Figure S5. Phosphorescence decay of PtTBTP film and PtTBTP/$DPP(PhCl)_2$ film (1 w/w% sensitizer). 13

Table S2. Fitting results of phosphorescence decay in *Figure S5*. 14

2.4 TTA control experiments for bare film 14

Figure S6. Time resolved emission decay of pristine $DPP(PhCl)_2$ film (prompt emission, λ_{ex}=475 nm) and upconverted emission of PtTBTP/$DPP(PhCl)_2$ film (delayed emission, λ_{ex}=613 nm). 14

Figure S7. Emission spectra of a pristine $DPP(PhCl)_2$ film when excited by a Nd:YAG laser-OPO at 490 nm (absorption maximum of annihilator) or 613 nm (absorption maximum of PtTBTP). 15

Figure S8. Emission spectrum of the cavity with only $DPP(PhCl)_2$ inside, and the sample is excited as 613 nm from the Nd:YAG laser and detected at the ICCD. 15

2.5 Polaritonic emission 16

Figure S9. Angle resolved emission of representative cavities when exciting at the maximum of their $|P^+\rangle$ peaks. Note that no polariton emission could be seen from Cav8. 16

Figure S10. Simulated electric field intensity distribution inside Cav7 by a transfer matrix approach. 16

2.6 Purcell effect of the cavity 17

Figure S11. The resonance frequency of Cav7. 17

2.7 Sensitized TTA emission of cavities 18

Figure S12. Sensitized TTA emission of bare film and Cav7 at ambient condition and in a cryostat with a N_2 atmosphere. 18

2.8 Transient absorption analysis of DPP(PhCl)$_2$. 19

Figure S13. Transient absorption spectra of (a) 4 μM PtTBTP and 100 μM DPP(PhCl)$_2$ in toluene; (b) PtTBTP/DPP(PhCl)$_2$ film (S-A film) when excited at 613 nm. 19

Figure S14. (a) Transient absorption decay at 485 nm of 4 μM PtTBTP and 1 mM perylene in toluene solution; (b) Transient absorption decay at 517 nm of 4 μM PtTBTP and 100 μM DPP(PhCl)$_2$ in toluene solution. 20

Table S3. Fitting results of transient absorption decays in *Figure S14*. 21

Figure S15. Initial transient absorption signal at 517 nm and calculated density of triplet DPP(PhCl)$_2$ in a sensitizer doped film (S-A film) as a function of excitation power. 21

2.9 Time resolved delayed fluorescence decay of bare film and cavities. 22

Figure S16. Time resolved delayed fluorescence decay of sensitizer doped film from 298 K to 113 K under different excitation intensities. 22

Figure S17. Time resolved delayed fluorescence decay of Cav7 from 298 K to 77 K under different excitation intensities. 22

Figure S18. Fractional contribution of exciton to polariton TTA (channel 1 in Figure 4c) and exciton to exciton TTA (channel 2 in Figure 4c) of the TTA emission of Cav7. 23

2.10 Spin statistic of TTA. 24

Scheme S3. The spin state of a singlet exciton, a triplet exciton and all the possible spin states of a triplet-triplet exciton pair. 24

2.11 Properties of the annihilator in aggregation. 25

Figure S19. Fluorescence quantum yield of DPP(PhCl)$_2$ at different concentrations in polystyrene matrix, when excited at 490 nm. 25

Figure S20. Transient absorption decay (λ_{ESA} = 537 nm) of DPP(PhCl)$_2$ at different concentrations in polystyrene matrix, when excited at 490 nm. 25

3 References 26

1 Experimental Section

1.1 Synthesis of the annihilator

All starting materials were purchased from Sigma-Aldrich Chemical Co. and used without further purification. All moisture- and oxygen-sensitive reactions were carried out using Schlenk techniques in oven-dried glassware. Solvents used for moisture and oxygen-sensitive reactions were dried using an MBraun MB SPS-800 solvent purification system and, if necessary, degassed by freeze-pump-thaw cycles and stored over 4-Å molecular sieves under argon atmosphere. Flash chromatography was performed using a Teledyne CombiFlash EZ prep and normal-phase silica. ^{1}H NMR (nuclear magnetic resonance) spectra were recorded on a Varian spectrometer at 400 MHz, J-coupling values are given in hertz, and chemical shifts are given in parts per million using tetramethysilane, with 0.00 ppm as an internal standard.

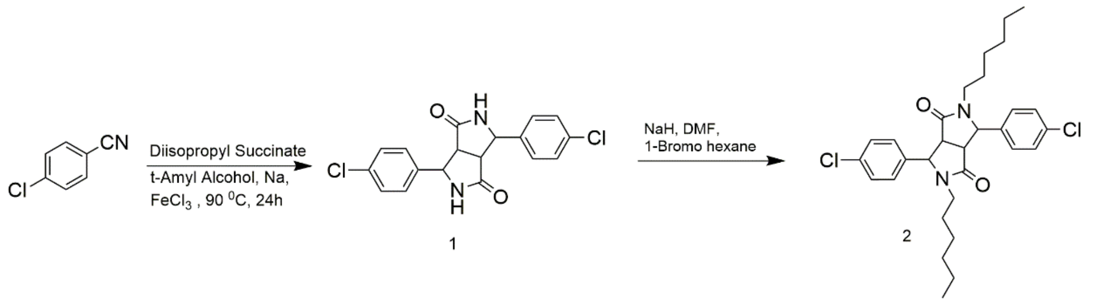

Scheme S1. Synthetic procedure of DPP(PhCl)$_2$.

3,6-bis(4-chlorophenyl)pyrrolo[3,4-c]pyrrole-1,4(2H,5H)-dione [DPP(PhCl)$_2$]: Synthesis of DPP(PhCl)$_2$ was performed following a previously reported procedure.[1] Sodium (1.25 g, 54.9 mmol) and $FeCl_3$ (0.06 g) was added to dry *t*-amyl alcohol (25 ml) at room temperature under Ar conditions, and the mixture was stirred for one hour at 90 °C. After the sodium was completely dissolved, the solution was cooled to 50 °C and 4-chlorobenzonitrile (3.77 g, 27.45 mmol) was added. The mixture was heated again to 90 °C and diisopropyl succinate (2.224 g, 11 mmol) in 10 ml of *t*-amyl alcohol was added drop wise. The mixture was stirred at 90 °C for 24 hr. Afterwards the mixture was cooled to 50 °C and acetic acid (11.5 ml) was added drop wise and the mixture was allowed to refluxed at 120 °C for 30 minutes. The reaction mixture was cooled down to room temperature and filtered. The filtered dark red

solid was washed several times with hot water and hot methanol, dried at 80 °C and directly used in next step without further purification (yield: 2.18 g, yield 44%).

2,5-Dihexyl-3,6-bis(4-chlorophenyl)pyrrolo[3,4-c]pyrrole-1,4-dione [2]: The compound 1 (1.8 g, 5.03 mmol) was added to 100 mL DMF in an oven-dried 250-mL one-necked flask, then NaH (0.48g, 20 mmol) (60% dispersion in mineral oil) was added portion wise. The mixture were stirred for 1 h at room temperature, followed by dropwise addition of 1-bromohexane (8.25 g, 50 mmol) in 25 mL of DMF. The mixture was kept for an additional 30h at room temperature and then filtered. The resulting solid was washed with 30 mL of chloroform, and the organic phase was washed with water (2*30mL) and brine (2*30mL). The combined organic phases was evaporated under reduced pressure and the residue was purified by silica-gel column chromatography, to give an orange-red powder (1.12 g, yield 50%). ^{1}H NMR (CDCl3;400MHz), δ(ppm): 0.84 (t, *J*=6.8Hz, 6H), 1.14–1.27(m, 12H), 1.50-1.58 (m, 4H), 3.72 (t, *J*=7.6Hz, 4H), 7.50 (m, 4H), 7.75 (m, 4H). Recorded data are in accordance with the literature.[1]

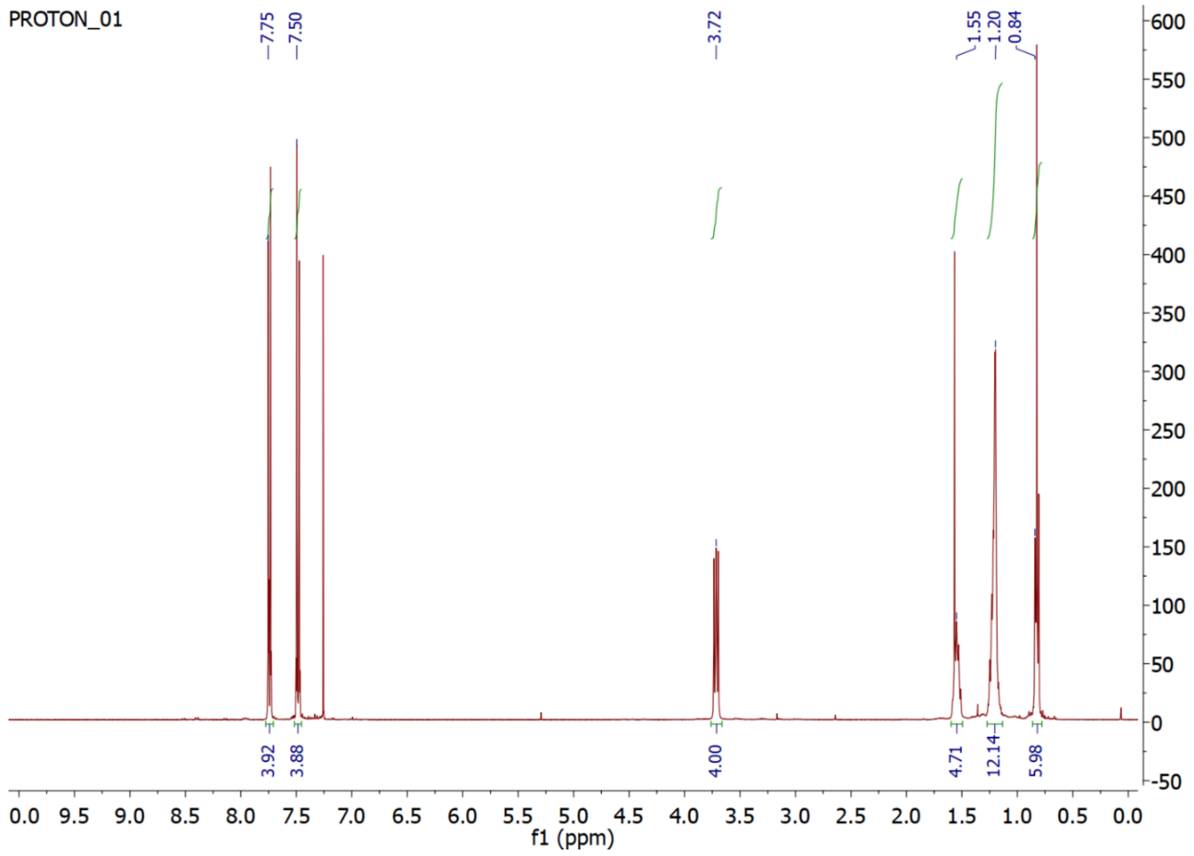

Figure S1. 1H NMR spectrum of DPP(PhCl)$_2$.

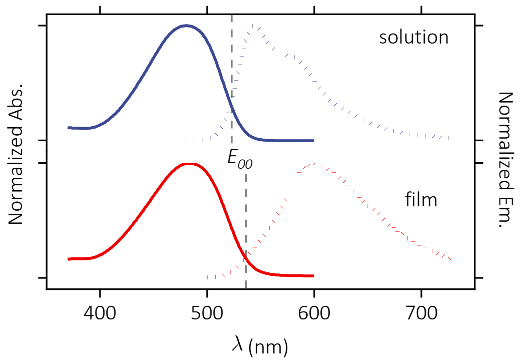

Figure S2. Molecular photophysics from dilute solution to film. Normalized absorption and emission of 10^{-5} M DPP(PhCl)$_2$ in toluene solution, and in a pristine DPP(PhCl)$_2$ film.

1.2 Film preparation

Glass substrates (1cm×1cm) were immersed in alkaline solution (0.5% of Hellmanex solution) and sonicated for 15 min. The substrates were then sonicated in water and ethanol for 1 h, successively. The cleaned glass substrates were finally dried in an oven. All film samples were deposited on the pre-cleaned glass substrates by spin-coating (Laurell Technologies WS6).

20 wt% DPP(PhCl)$_2$ film: 7.2 mg DPP(PhCl)$_2$ and 28.8 mg polystyrene were dissolved in 1 mL toluene, and 100 μL of the solution was deposited on a clean glass at 1800 rpm.

Pristine DPP(PhCl)$_2$ film: 36 mg DPP(PhCl)$_2$ was dissolved in 1 mL toluene, and 100 μL of the solution was deposited on a clean glass at 1800 rpm.

PtTBTP/DPP(PhCl)$_2$ films (S-A film): 0.36 mg PtTBTP and 36 mg DPP(PhCl)$_2$ were dissolved in 1 mL toluene, and 100 μL of the solution was deposited on a clean glass at various rpm (Table S1).

1.3 Cavity preparation

The Fabry–Pérot cavities were built on 1cm×1cm clean glass substrates. The 150 nm bottom mirrors were deposited by vacuum sputtering (HEX, Korvus Technologies). The molecular films were then deposited on the bottom mirrors by spin-coating (Laurell Technologies WS6). The 30 nm top mirrors were deposited on the top of molecular layer by vacuum sputtering. The detailed parameters for different cavities are listed as below:

Table S1. Parameters for the cavities in this work.

Sample	Solutions for preparation		Spin-Coating speed
	PtTBTP	DPP(PhCl)$_2$	(rpm)
Cav1	0.25 mg/mL	25 mg/mL	2000
Cav2	0.36 mg/mL	36 mg/mL	3600
Cav3	0.36 mg/mL	36 mg/mL	2400
Cav4	0.36 mg/mL	36 mg/mL	1900
Cav5	0.36 mg/mL	36 mg/mL	1800
Cav6	0.36 mg/mL	36 mg/mL	1800
Cav7	0.36 mg/mL	36 mg/mL	1800
Cav8	0.40 mg/mL	40 mg/mL	1000

1.4 Modelling the angle-resolved reflectivity.

The angle-resolved reflectivity of cavities were fitted by a coupled oscillator model (*eq.1*). The energy dispersion of cavities (E_c) are given by:

$$E_c(\theta) = \frac{E_c(0)}{\sqrt{1 - \left(\frac{\sin\theta}{n_{eff}}\right)^2}} \quad (eq. S1)$$

where θ is the incident angle, and η_{eff} is the refractive index inside the cavity. The energy dispersion of the polariton states were then obtained from the eigenvectors of the Hamiltonian in *eq. 1*.

1.5 Steady state absorption, reflection and emission spectroscopy.

Absorption spectra for solution samples and films were recorded on a PerkinElmer LAMBDA 950 spectrometer. Angle resolved reflectivity spectra of cavities were recorded on a PerkinElmer LAMBDA 950 spectrometer with a universal reflectance accessory.

Steady state emission of solution and films were measured on an Edinburgh Instruments FLS 1000 spectrofluorometer with a Xenon lamp as excitation source. Angular resolved emission spectra of cavities were measured on the same instrument using a fiber induced angle-resolved platform.

1.6 Transfer matrix method.

The absorption inside the bare film was assumed to follow the Beer-Lambert law. The electric field profile of the representative cavity Cav7 was obtained using a standard transfer matrix method.[2] The cavity consists of a 30 nm Ag top layer, a molecular layer, and a 150 nm bottom Ag layer. The thickness of Ag layers were estimated by a QCM crystal inside the sputterer, which in turn was calibrated using a profilometer. The complex refractive index of Ag was obtained from P. B. Johnson and R. W. Christy's measurement with interpolation.[3] The complex refractive index of the molecular layer was obtained using the Kramers–Kronig relations.[4] The imaginary part of the molecular refractive index (extinction coefficient) was obtained from the absorption spectrum of the bare film. The real part of the refractive was then calculated from the extinction coefficient by the Kramers–Kronig relations.

In the simulation of the transfer matrix, the layer thickness were treated as the fitting values. By comparing the measured and the simulated reflectance spectra, we determined the exact thickness of the top mirror, molecular layer, and the bottom mirror in Cav7 to 31 nm, 121 nm, and 150 nm, respectively.

The total electric field $E(x)$ inside the cavity at the layer position x along the cavity depth was described as the superposition of the forward and backward propagating plane waves[5]:

$$E(x) = E_F e^{ik_F x} + E_B e^{ik_B x} (eq. S2)$$

where E_F and E_B are the forward and backward amplitudes, and k_F and k_B are the forward and backward wave vectors. The power density of a plane wave inside the cavity was given by the real part of the Poynting vector:

$$I(x) = \langle \boldsymbol{S}(x) \rangle = Re(n) \frac{|E(x)|^2}{2Z_0} (eq. S3)$$

where Z_0 is the impedance of vacuum.

From the transfer matrix method, E(x), is obtained. The total absorbance, transmittance and reflectance of the system adheres to the conservation of photon flux. The internal light absorbance of the thickness from depth x_1 to depth x_2 was calculated by integrating the light intensity[6]:

$$A = \int_{x_1}^{x_2} \alpha(x) I(x) dx \; (eq. S4)$$

The triplet transfer from the sensitizer to the annihilator is fast and efficient, due to the excess concentration of ground state annihilators in the molecular layer. We thus assumed that the absorbed photons by the sensitizer will all contribute to the formation of triplet annihilator excitons. This assumption goes well in hand with experimental data, showing a

reduction of the sensitizer lifetime with one order of magnitude in presence of the annihilator.

1.7 TTA photon upconversion emission.

TTA photon upconversion experiments of bare films and cavities were carried out on an Edinburgh Instruments LP 980 transient absorption spectrometer (Scheme S2). A Spectra-Physics Nd:YAG 532 nm laser (pulse width 7 ns) coupled to a Spectra-Physics primoscan optical parametric oscillator (OPO) was used as excitation source. An Andor iSatr 334 fast gated intensified CCD was used as the detector. All spectra were recorded with a 4 ms gate width to cover the whole TTA signal and a 500 ns gate delay to avoid scattering and prompt emission. All samples were placed inside an Oxford Instrument Optistat DN-V cryostat with N_2 atmosphere. The temperature of the samples were controlled by a MercuryiTC temperature controller.

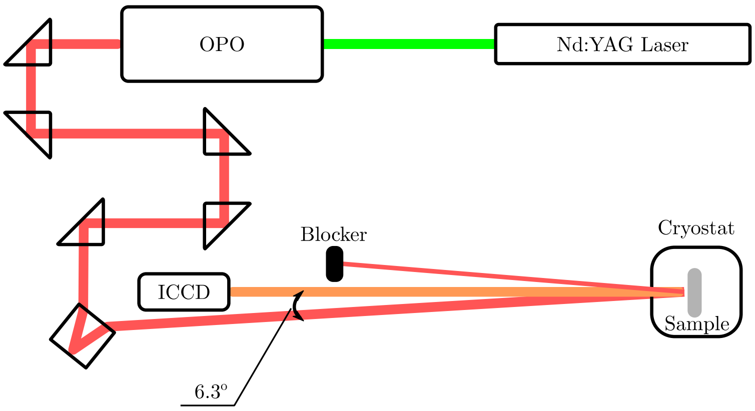

Scheme S2. Schematic illustration of experimental setup for the steady-state TTA photon upconversion measurements.

1.8 Time resolved photoluminescence spectroscopy.

Time resolved photoluminescence decay of the bare film and cavities were recorded in an N_2 atmosphere provided by a cryostat on an Edinburgh Instruments FLS 1000 spectrofluorometer in Multi-Channel Scaling (MCS) mode. A Spectra-Physics Nd:YAG laser (pulse width 7 ns) coupled to a Spectra-Physics primoscan optical parametric oscillator (OPO) was used as the excitation source. A photomultiplier tube (PMT) was used as the detector. Time resolved delayed fluorescence decays in bare films and cavities follow eq. 2-3.

We measured the time resolved emission at different temperatures. The excitation power (I_{ex}) was recorded to calculate the initial density of the triplet annihilator (ρ_0) after excitation (from Figure S14-16). Cavities absorb less light due to the strongly reflecting top mirror, and the initial triplet densities was calibrated using the cavity reflectance spectra (Figure 1a).[7] We measured the time resolved emission under 2 different excitation powers at every temperature. The kinetic parameters (γ_{TTA} and k_T) were calculated by global fitting from the 2 measurements.

1.9 Transient absorption spectroscopy.

Transient absorption spectra of solution and the bare film were recorded on an Edinburgh Instruments LP 980 transient absorption spectrometer with a Nd:YAG laser coupled to a OPO as the excitation source under N_2 atmosphere.

2 Supporting Experimental Results

2.1 Properties of cavities

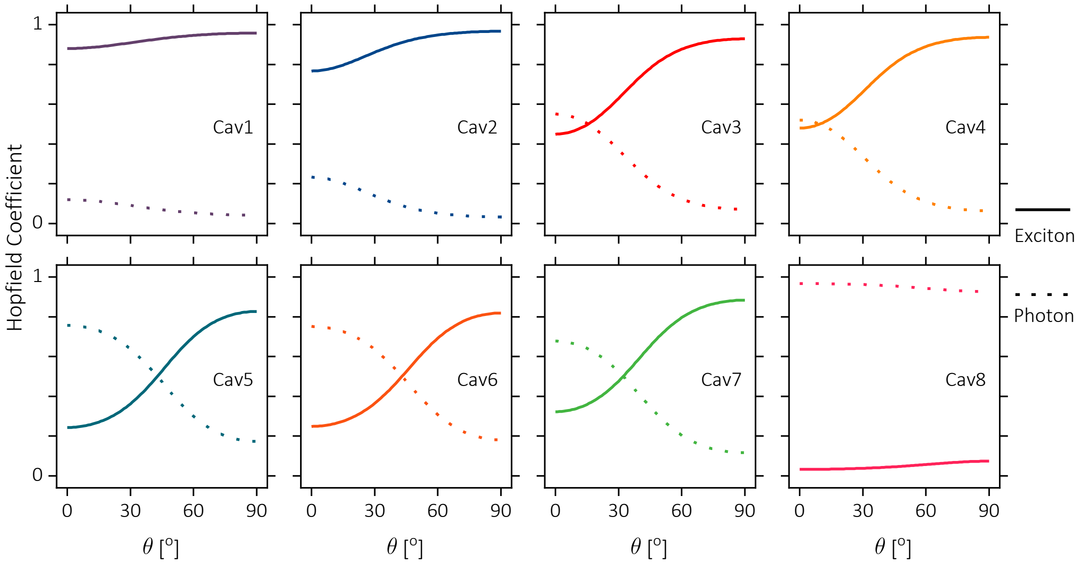

Figure S3. Hopfield coefficient of |P⁻⟩ of the cavities.

2.2 Information of the sensitizer

The sensitizer platinum tetra-benzo-tetra-phenyl-porphyrin (PtTBTP) was purchased from PorphyChem.

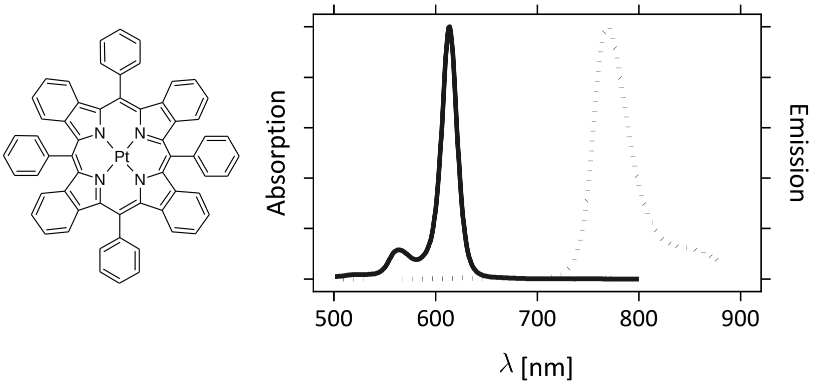

Figure S4. Molecular structure and absorption and emission spectrum of PtTBTP in toluene.

2.3 Triplet-triplet energy transfer

Triplet energy transfer from PtTBTP to $DPP(PhCl)_2$ can be confirmed from the quenched phosphorescence of PtTBTP in solid films. Both samples were excited at 613 nm and the phosphorescence was detected at 767 nm. Phosphorescence decays were analyzed with a stretched exponential decay model (*eq. S2*):

$$I = \sum A_i exp\left(-(t/\tau_i)^{\beta_i}\right) \quad (eq. S5)$$

where *I* is the decay amplitude over time, *t* is the decay time, A_i is pre-exponential factor of decay component *i*, τ_i is decay lifetime of decay component *i*, β_i is compressed exponential function. The mean relaxation time of the phosphorescence in film samples are calculated by the following equation (*eq. S3*):

$$\langle\tau\rangle = \int_0^\infty dt e^{-(t/\tau)^\beta} \quad (eq. S6)$$

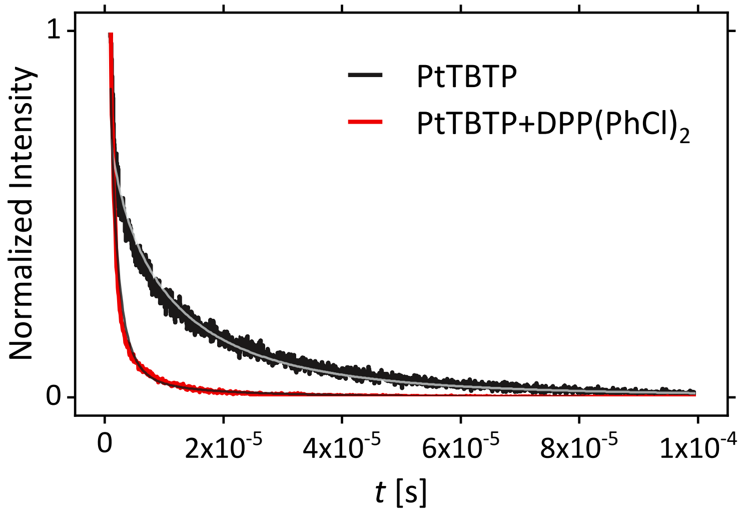

Figure S5. Phosphorescence decay of PtTBTP film and PtTBTP/$DPP(PhCl)_2$ film (1 w/w% sensitizer).

Table S2. Fitting results of phosphorescence decay in *Figure S5*.

Sample	τ_1 [μs]	A_1	β_1	$<\tau_1>$ [μs]	τ_2 [μs]	A_2	β_2	$<\tau_2>$ [μs]	$\tau_{average}$ [μs]
PtTBTP film	8.960	0.912	0.634	12.59					12.59
PtTBTP/DPP(PhCl)$_2$ film	0.566	3.202	0.663	0.755	8.659	0.070	0.634	12.17	3.735

2.4 TTA control experiments for bare film

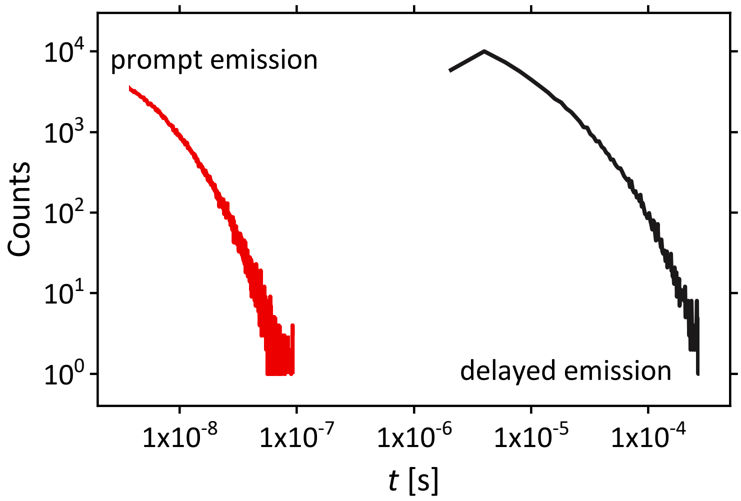

Figure S6. Time resolved emission decay of pristine DPP(PhCl)$_2$ film (prompt emission, λ_{ex}=475 nm) and upconverted emission of PtTBTP/DPP(PhCl)$_2$ film (delayed emission, λ_{ex}=613 nm).

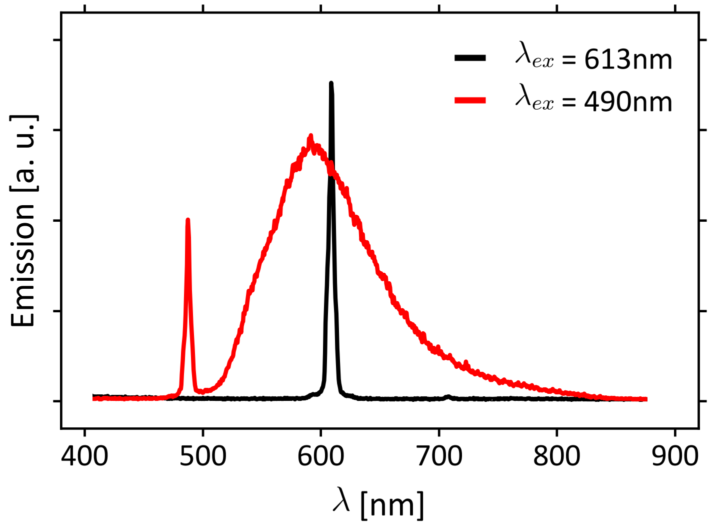

Figure S7. Emission spectra of a pristine DPP(PhCl)$_2$ film when excited by a Nd:YAG laser-OPO at 490 nm (absorption maximum of annihilator) or 613 nm (absorption maximum of PtTBTP).

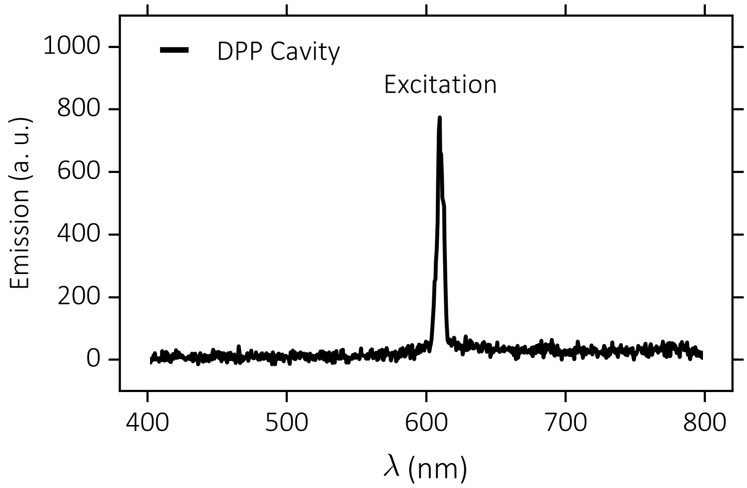

Figure S8. Emission spectrum of the cavity with only DPP(PhCl)$_2$ inside, and the sample is excited as 613 nm from the Nd:YAG laser and detected at the ICCD.

2.5 Polaritonic emission

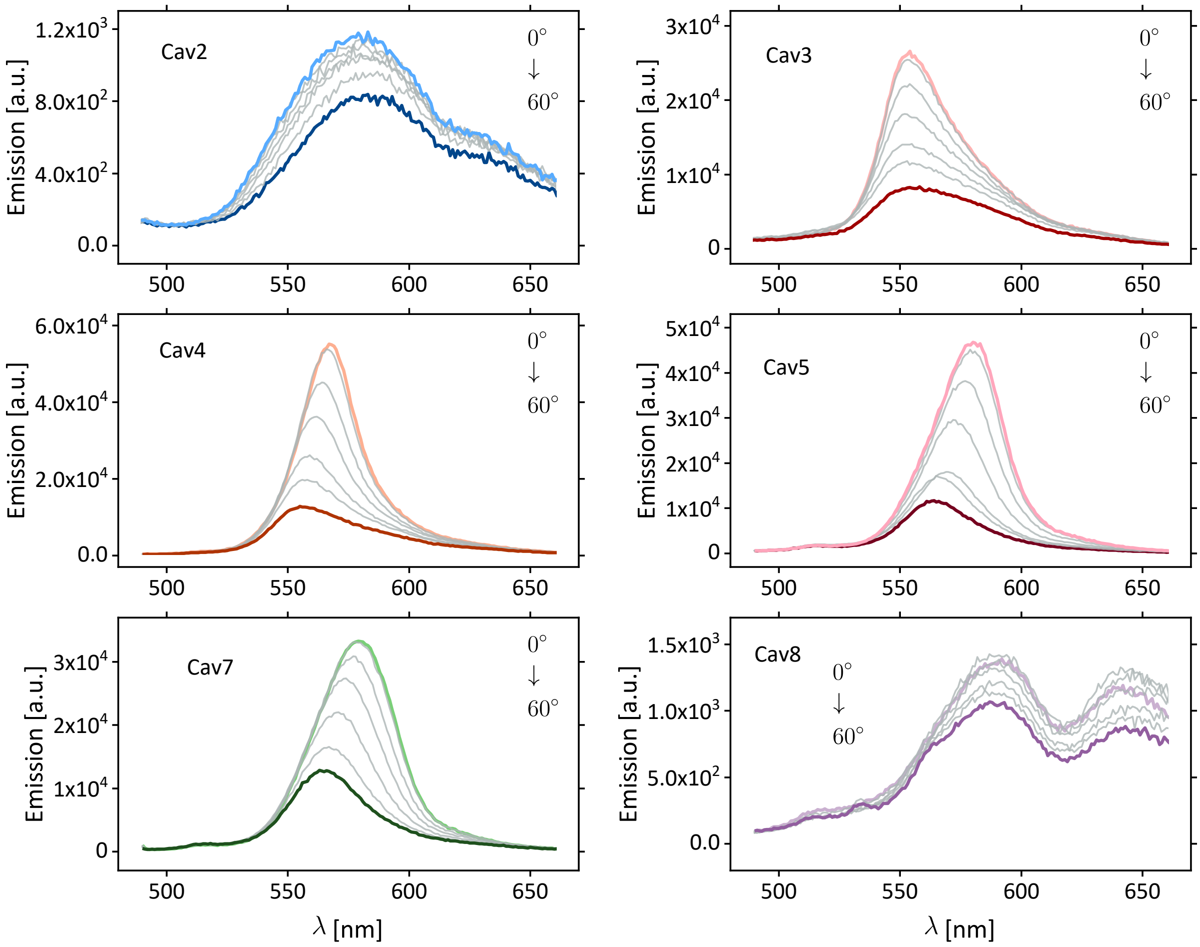

Figure S9. Angle resolved emission of representative cavities when exciting at the maximum of their $|P^{+}\rangle$ peaks. Note that no polariton emission could be seen from Cav8.

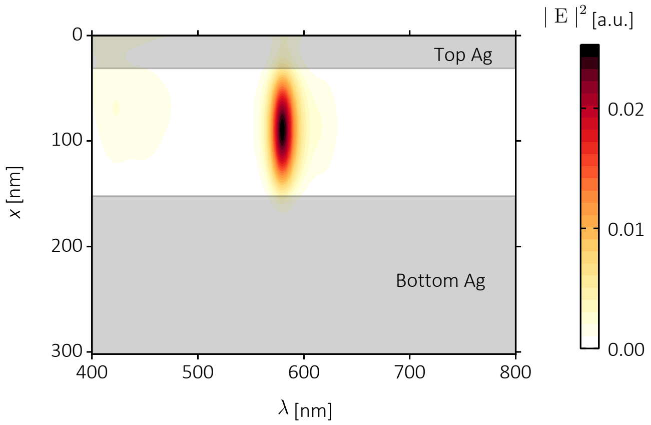

Figure S10. Simulated electric field intensity distribution inside Cav7 by a transfer matrix approach.

2.6 Purcell effect of the cavity

The enhanced magnitude of the spontaneous emission of an emitter located in an optical cavity can be described by the Purcell factor (Fp). The Purcell factor of the Cav7 was calculated by the following equation:

$$F_P = \frac{3}{4\pi^2}\left(\frac{\lambda}{n}\right)^3\left(\frac{Q}{V}\right) \quad (eq.S7)$$

where *n* is the refractive index, *λ* is the wavelength, *Q* is the quality factor of the cavity, and *V* is the mode volume of the cavity. The quality factor *Q* is defined as the frequency-to-bandwidth ratio of the cavity:

$$Q = \frac{\omega}{\Delta\omega} \quad (eq.S8)$$

where *ω* is the resonant frequency of the cavity, and *Δω* is the bandwidth of the photonic peak. The Q for Cav7 was calculated to be 27 (Figure S11).

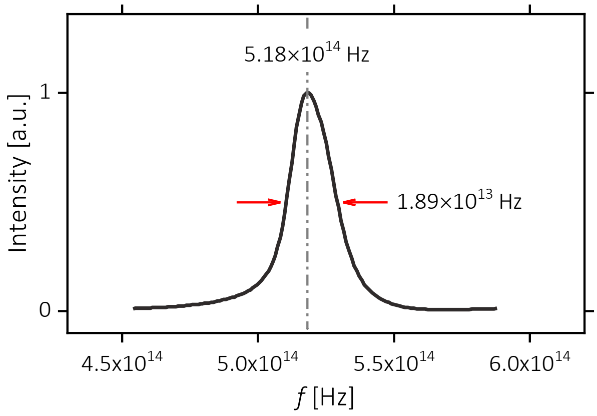

Figure S11. The resonance frequency of Cav7.

The mode volume of the cavity mode volume can be described by the following equation:

$$V = \frac{\iiint \varepsilon|E|^2 dxdydz}{(\varepsilon|E|^2)_{max}} \quad (eq.S9)$$

where *ε* is the dielectric constant, and *E* is the electric field intensity inside the cavity (shown in Figure S10). The laser spot diameter is 5 mm, and the cavity thickness is calculated from

the transfer matrix approach. We then calculated the mode volume of the cavity to be 1.71 $\times 10^{-12}$ m^3. The *Fp* of Cav7 was then calculated to be 8.79×10^{-8}, which is negligible as compared to the TTA enhancement. We can therefore exclude the Purcell effect as the main reason for the efficient TTA emission in the cavities we presented.

2.7 Sensitized TTA emission of cavities

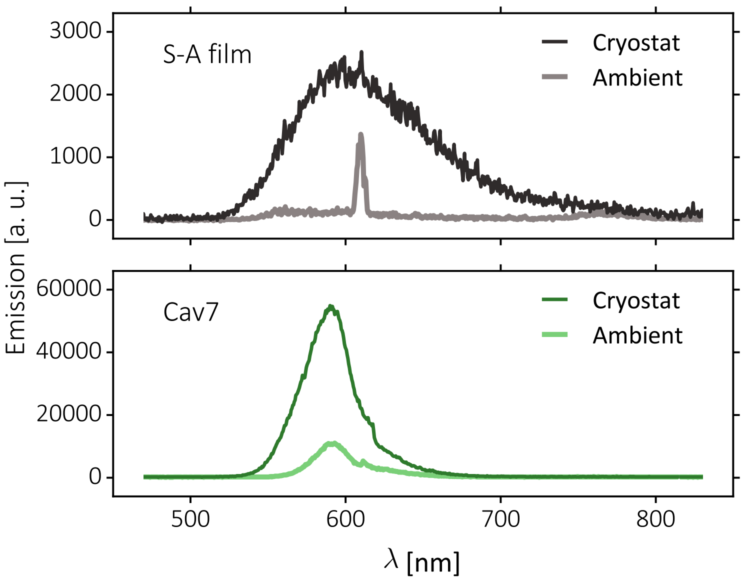

Figure S12. Sensitized TTA emission of bare film and Cav7 at ambient condition and in a cryostat with a N_2 atmosphere.

2.8 Transient absorption analysis of DPP(PhCl)$_2$.

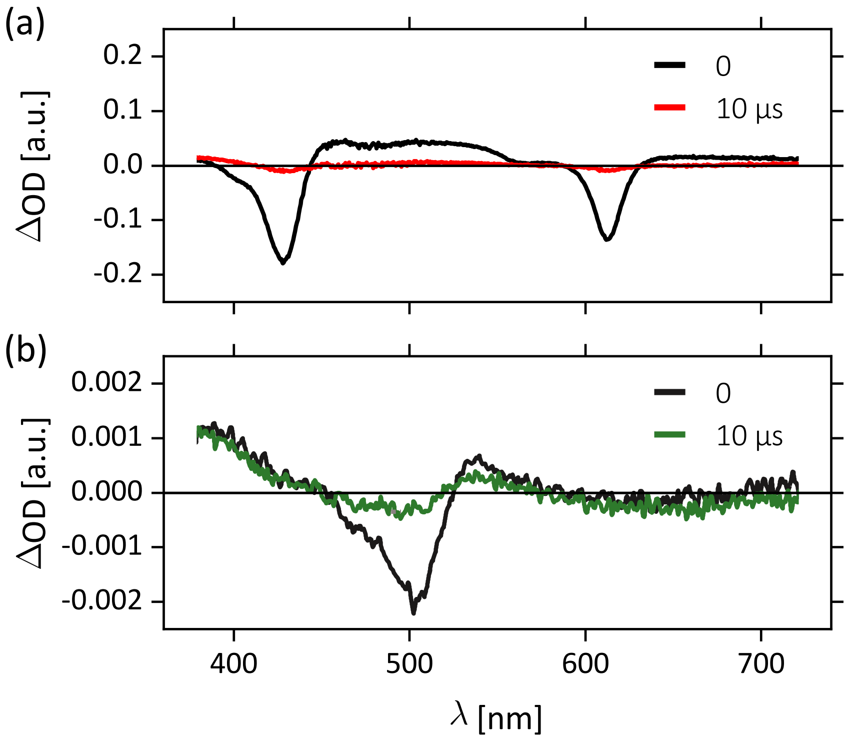

Figure S13. Transient absorption spectra of (a) 4 μM PtTBTP and 100 μM DPP(PhCl)$_2$ in toluene; (b) PtTBTP/DPP(PhCl)$_2$ film (S-A film) when excited at 613 nm.

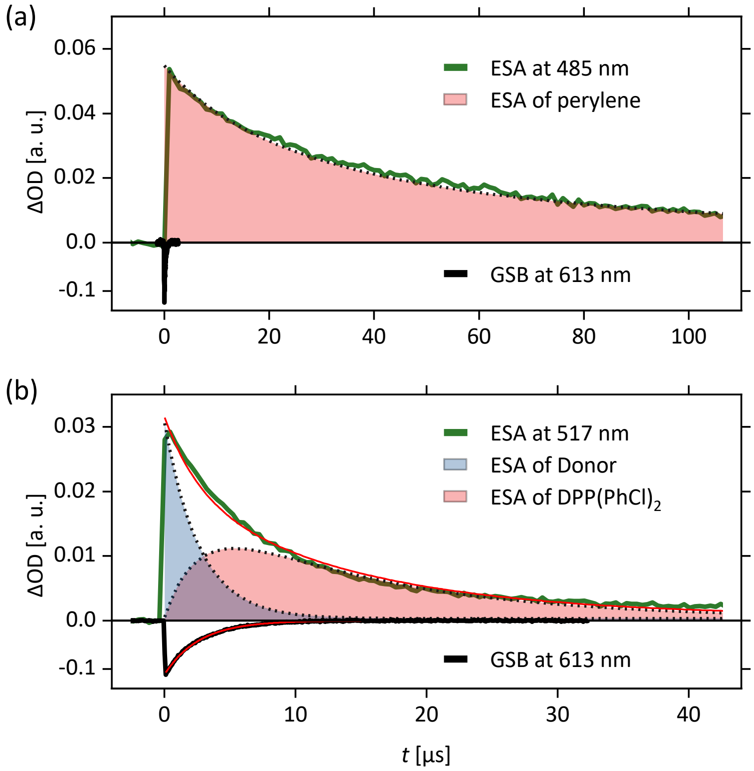

Figure S14. (a) Transient absorption decay at 485 nm of 4 μM PtTBTP and 1 mM perylene in toluene solution; (b) Transient absorption decay at 517 nm of 4 μM PtTBTP and 100 μM $DPP(PhCl)_2$ in toluene solution.

Perylene is an efficient TTA annihilator. We used perylene as a reference to calculate the molar absorptivity of the $DPP(PhCl)_2$ T_1 to T_n transition.[8,9] The transient absorption decay of triplet perylene can be fitted by the TTA kinetic model:

$$\begin{cases} \frac{\mathrm{d}[\mathrm{T}_A]}{\mathrm{dt}} = -\frac{[\mathrm{T}_A]}{\tau} - k_{TTA}[\mathrm{T}_A]^2 \\ \Delta OD = \varepsilon l[\mathrm{T}_A] \end{cases} \quad (eq.S10$$

where $[T_A]$ is the concentration of perylene in the triplet excited state, τ is the intrinsic decay lifetime of the triplet excited state in solution, k_{TTA} is the TTA rate constant in solution (note, we call this parameter γ_{TTA} in the solid state as to differentiate between TTA in solution and solid state), l is the light path length of the cuvette, and ε is the molar absorptivity of the triplet species at the detection wavelength.

$DPP(PhCl)_2$ cannot undergo TTA in solution due to endothermicity of the process, and the transient absorption decay of triplet $DPP(PhCl)_2$ can therefore be fitted by an exponential decay model:

$$\begin{cases} \frac{d[T_D]}{dt} = -k_{ph}[T_D] - k_{TET}[T_D][A] \\ \frac{d[T_A]}{dt} = k_{TET}[T_D][A] - \frac{[T_A]}{\tau} \\ \Delta OD = \varepsilon l [T_A] \end{cases} \quad (eq.S11)$$

where $[T_D]$ is the concentration of donor PtTBTP in the triplet excited state, k_{ph} is the phosphorescence decay lifetime of donor PtTBTP with absence of annihilator, [A] is the concentration of acceptor $DPP(PhCl)_2$ in the ground state, $[T_A]$ is the concentration of acceptor $DPP(PhCl)_2$ in the triplet excited state, τ is the intrinsic decay lifetime of the triplet excited states in solution, and k_{TET} is the rate constant of triplet triplet energy transfer.

Table S3. Fitting results of transient absorption decays in *Figure S14*.

Sample	τ [μs]	ΔOD_{max}	k_{TTA} [$M^{-1}s^{-1}$]	$\varepsilon(T_1\text{-}T_n)$ [$cm^{-1}M^{-1}$]
PtTBTP+Perylene	106	5.0×10^{-2}	6.2×10^{9}	13400 (λ=485 nm)[8,9]
PtTBTP+$DPP(PhCl)_2$	13.8	1.9×10^{-2}	NA	5063 (λ=517 nm)

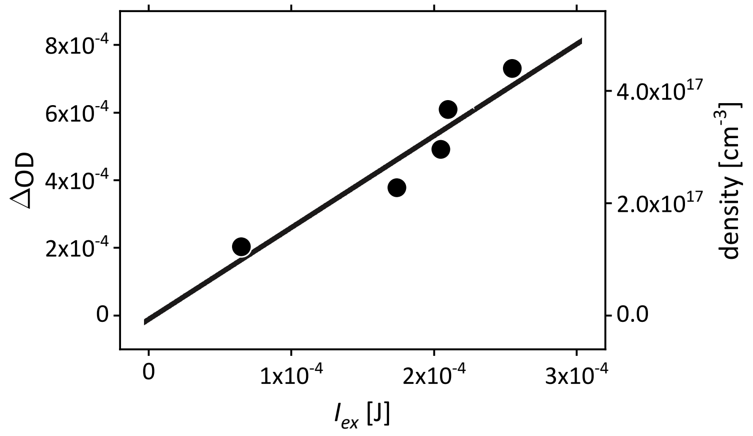

Figure S15. Initial transient absorption signal at 517 nm and calculated density of triplet $DPP(PhCl)_2$ in a sensitizer doped film (S-A film) as a function of excitation power.

2.9 Time resolved delayed fluorescence decay of bare film and cavities.

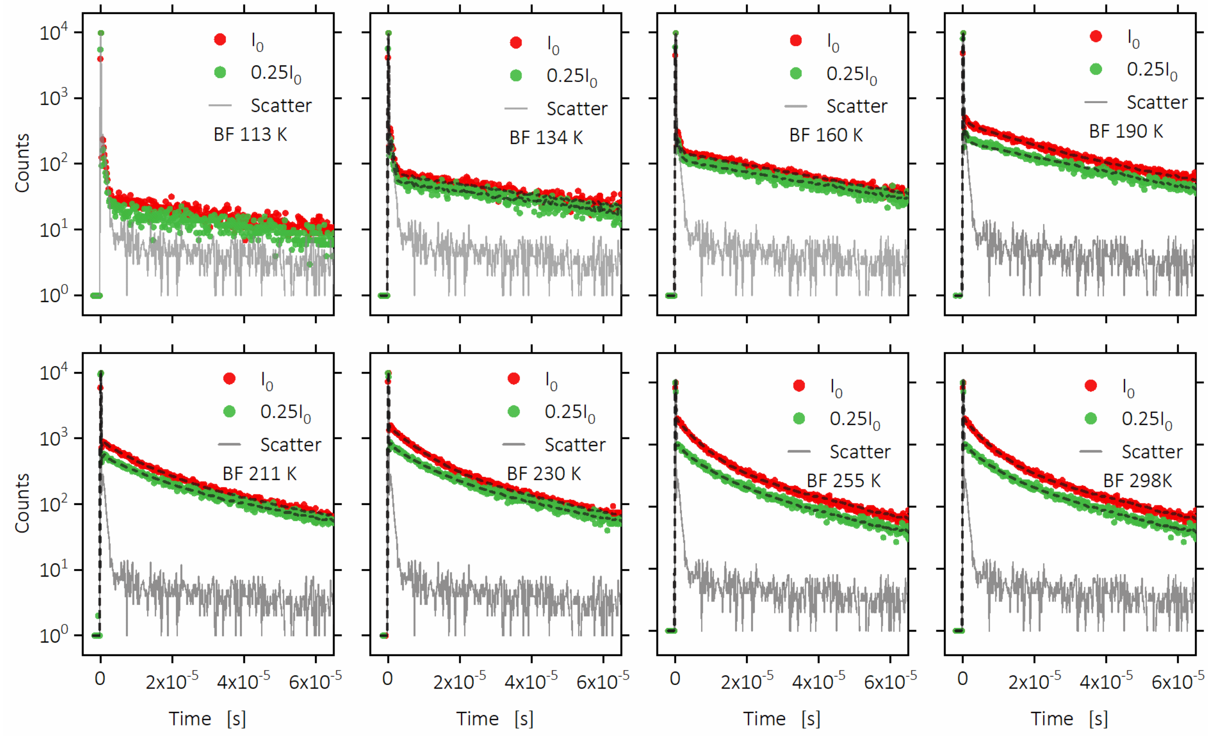

Figure S16. Time resolved delayed fluorescence decay of sensitizer doped film from 298 K to 113 K under different excitation intensities.

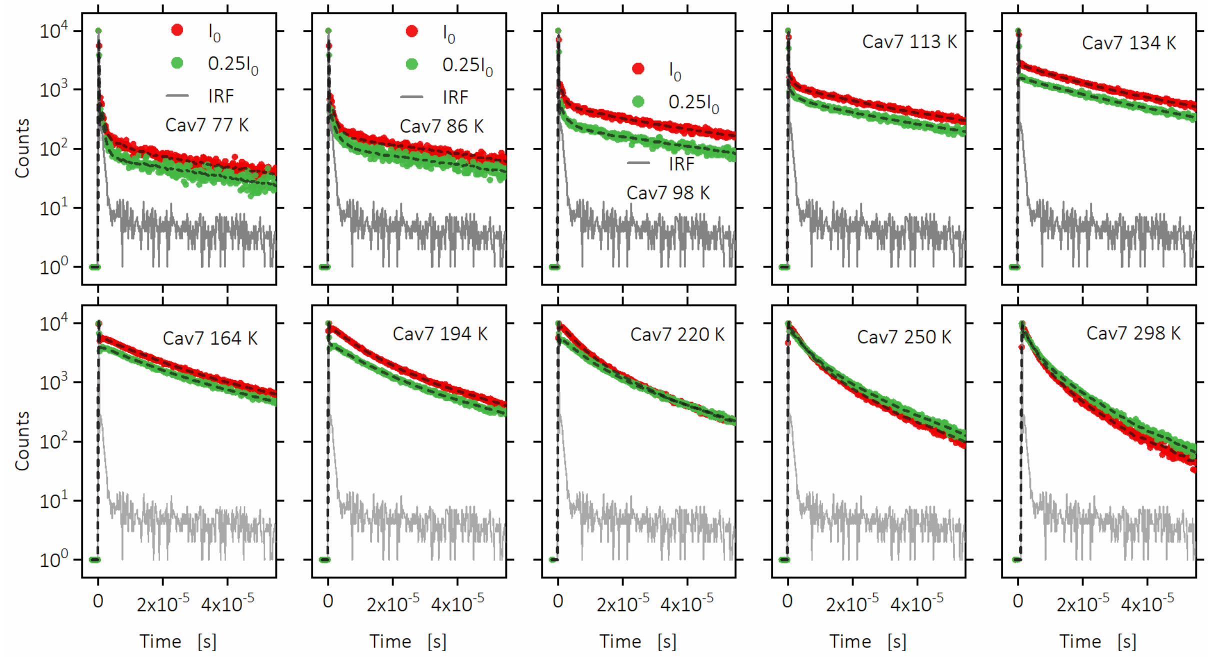

Figure S17. Time resolved delayed fluorescence decay of Cav7 from 298 K to 77 K under different excitation intensities.

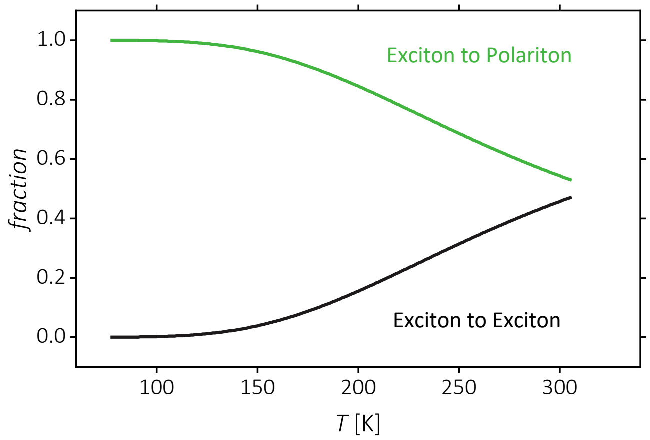

Figure S18. Fractional contribution of exciton to polariton TTA (channel 1 in Figure 4c) and exciton to exciton TTA (channel 2 in Figure 4c) of the TTA emission of Cav7.

2.10 Spin statistic of TTA.

A triplet pair complex encounter has 9 types of spin configurations. The singlet encounter complex ^{1}(TT) can convert into one singlet excited state and one ground state molecule. The spin angular momentum is then conserved in TTA.

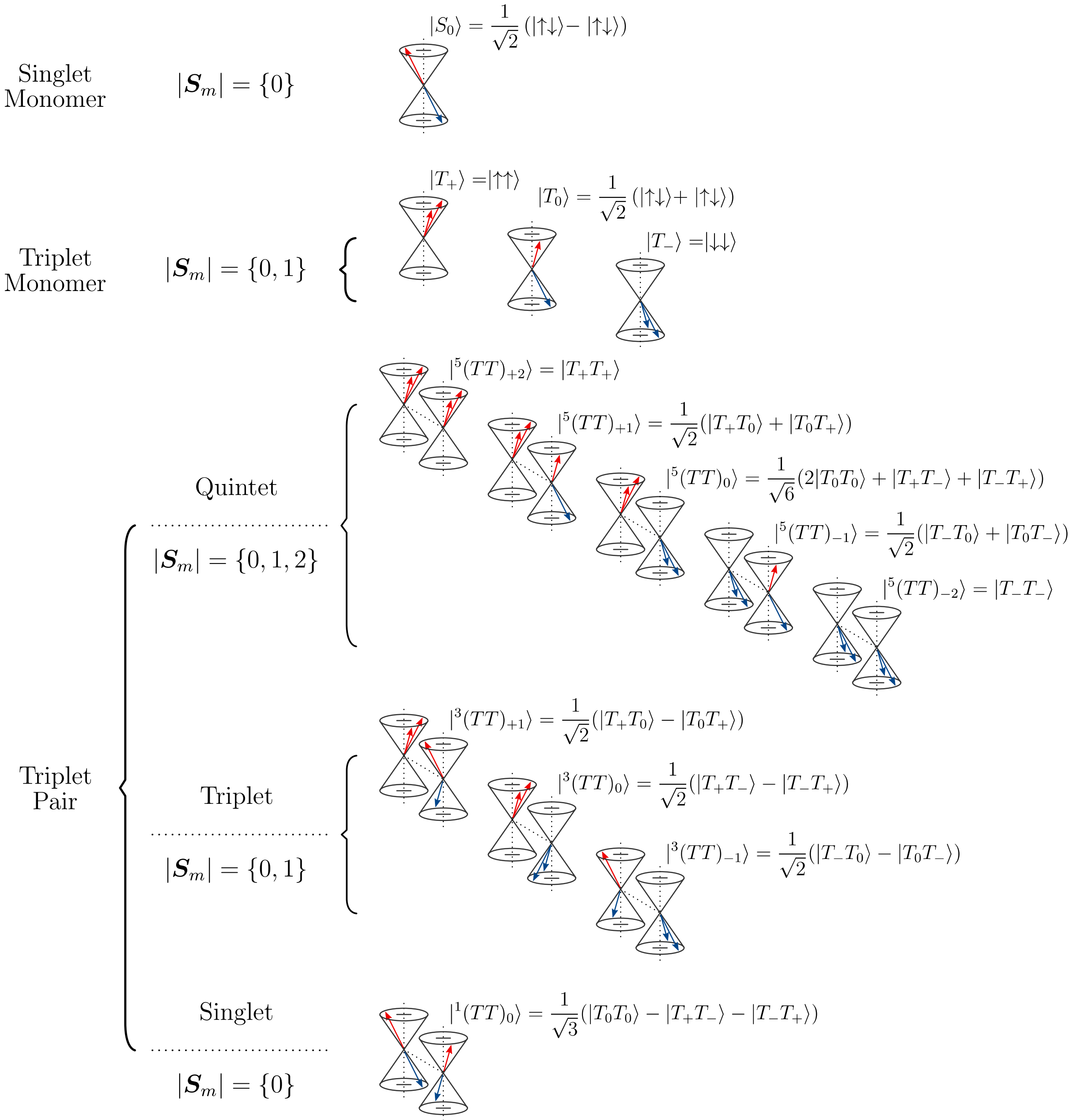

Scheme S3. The spin state of a singlet exciton, a triplet exciton and all the possible spin states of a triplet-triplet exciton pair.

2.11 Properties of the annihilator in aggregation.

The quantum yield of $DPP(PhCl)_2$ film increases with the concentration.

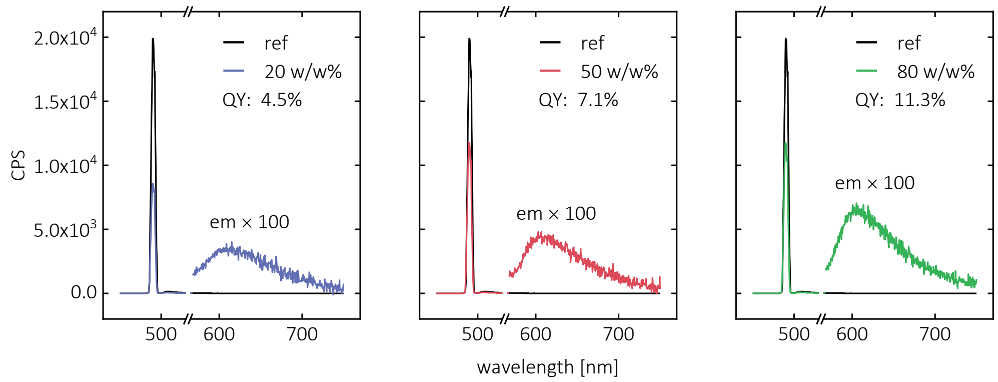

Figure S19. Fluorescence quantum yield of $DPP(PhCl)_2$ at different concentrations in polystyrene matrix, when excited at 490 nm.

The decay lifetime of the triplet state of $DPP(PhCl)_2$ in a polystyrene matrix is independent to the concentration of ground states molecules.

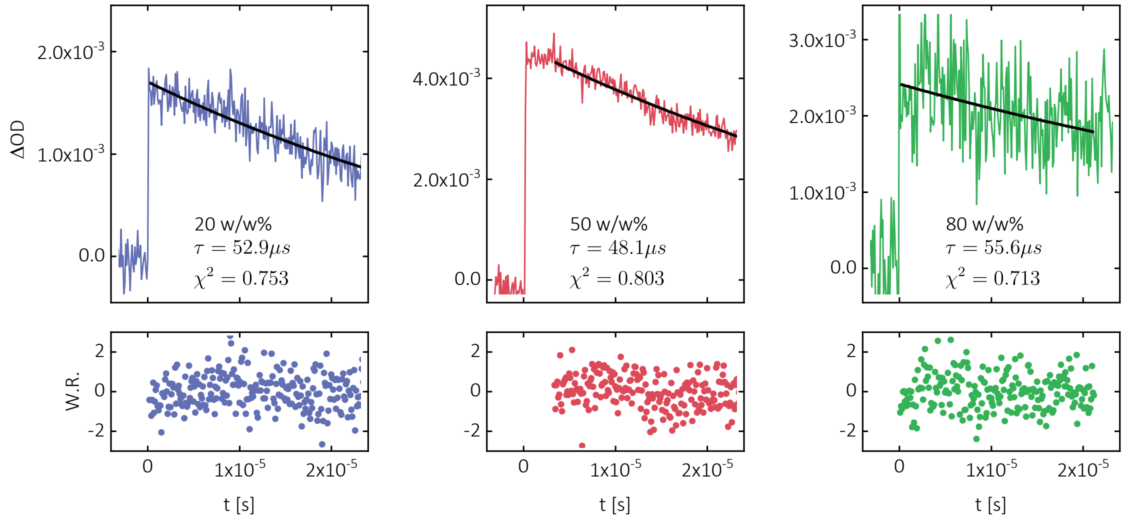

Figure S20. Transient absorption decay (λ_{ESA} = 537 nm) of $DPP(PhCl)_2$ at different concentrations in polystyrene matrix, when excited at 490 nm.

3 References

1 Pun, A. B., Campos, L. M. & Congreve, D. N. Tunable Emission from Triplet Fusion Upconversion in Diketopyrrolopyrroles. *J. Am. Chem. Soc.* **141**, 3777-3781, doi:10.1021/jacs.8b11796 (2019).

2 Byrnes, S. J. Multilayer optical calculations. arXiv:1603.02720 (2016). <https://ui.adsabs.harvard.edu/abs/2016arXiv160302720B>.

3 Johnson, P. B. & Christy, R. W. Optical Constants of the Noble Metals. *Phys. Rev. B* **6**, 4370-4379, doi:10.1103/PhysRevB.6.4370 (1972).

4 Lucarini, V., Saarinen, J. J., Peiponen, K. E. & Vartiainen, E. M. *Kramers-Kronig Relations in Optical Materials Research*. (Springer Berlin Heidelberg, 2005).

5 Ohta, K. & Ishida, H. Matrix formalism for calculation of electric field intensity of light in stratified multilayered films. *Appl. Opt.* **29**, 1952-1959, doi:10.1364/AO.29.001952 (1990).

6 Pettersson, L. A. A., Roman, L. S. & Inganäs, O. Modeling photocurrent action spectra of photovoltaic devices based on organic thin films. *J. Appl. Phys.* **86**, 487-496, doi:10.1063/1.370757 (1999).

7 Virgili, T. *et al.* Ultrafast polariton relaxation dynamics in an organic semiconductor microcavity. *Phys. Rev. B* **83**, 245309, doi:10.1103/PhysRevB.83.245309 (2011).

8 Carmichael, I., Helman, W. P. & Hug, G. L. Extinction Coefficients of Triplet–Triplet Absorption Spectra of Organic Molecules in Condensed Phases: A Least‐Squares Analysis. *J. Phys. Chem. Ref. Data* **16**, 239-260, doi:10.1063/1.555782 (1987).

9 Ye, C., Gray, V., Mårtensson, J. & Börjesson, K. Annihilation Versus Excimer Formation by the Triplet Pair in Triplet–Triplet Annihilation Photon Upconversion. *J. Am. Chem. Soc.* **141**, 9578-9584, doi:10.1021/jacs.9b02302 (2019).
